# The impact of ethnic self-identity on post-migration social integration of young rural to urban migrants in China: An interpretative phenomenological analysis

**DOI:** 10.1371/journal.pone.0353655

**Published:** 2026-07-16

**Authors:** Tianning Li

**Affiliations:** The Industrial Engineering College, Ningxia Vocational and Technical University (Ningxia Open University), Yichuan, Ningxia, China; Zhejiang Agriculture and Forestry University: Zhejiang A and F University, CHINA

## Abstract

**Background:**

Post-migration social integration is a pressing concern for young ethnic-minority migrants in urban China. This study examines how ethnic self-identity shapes this integration process from the migrant’s own lived perspectives. Using an interpretative phenomenological approach, we regard young migrants as subjects of meaning-making rather than objects to be measured. Ecological systems theory, social identity theory, and social cognitive models provide the analytical frameworks for understanding how ethnic identity is negotiated within destination-city contexts.

**Methods:**

We conducted 30 semi-structured interviews with ethnic migrant youths from Miao, Tujia, and Zangzu (Tibetan) groups who had relocated to Wuhan, Hubei Province. Data were analyzed using Interpretative Phenomenological Analysis, with cross-case patterning organized through thematic procedures.

**Results:**

Analysis identified five interconnected experiential dimensions of ethnic self-identity that influence social integration: ethnic self-categorization, ethnic versus local language proficiency, ethnic and cultural practices, perceived ethnicity- based discrimination and bicultural identity integration.

**Conclusion:**

Ethnic self-identity functions as a two-sided mechanism: it simultaneously provides psychological protection against discrimination and sustains ethnic group cohesion, yet also creates social boundaries that complicate host-community integration. These findings highlight the need for policies and interventions that address both identity-based support and structural barriers to integration.

## Introduction

Social integration of ethnic migrant youths in urban China is a pressing policy and scholarly concern. For young rural-to-urban migrants from ethnic minority groups, establishing meaningful social, economic, and psychological ties within their destination city represents a critical developmental challenge with implications for individual well-being, urban governance and social cohesion. Post migration social integration refers to the multi-dimensional process through which migrants establish meaningful social, economic, and psychological ties within their destination city in the aftermath of relocation. For young migrants from ethnic minorities, this process is shaped by how they negotiate their ethnic self-identity in the new urban environment. It encompasses accessing equitable employment, building social networks, navigating public services and developing a sense of belonging in the destination city.

A key factor shaping this integration is ethnic self-identity. Ethnic self-identity refers to an individual’s subjective affirmation in terms of membership to a particular ethnic group that incorporates a sense of belonging and the value attached to that group [1]. Kinket and Verkuyten conceptualized ethnic self-identification as encompassing a sense of belonging to the group, perceived similarity, identity salience, commitment, and participation in the group’s cultural practices [2]. In simple terms, it refers to how an individual label themselves and their ethnic group affiliation. However, in broader terms, it also encompasses various aspects, such as belongingness, commitment, a sense of shared values, and an attitude towards one’s own ethnic group [3]. As a key indicator of social integration for rural to urban migrants, self-identity reflects the degree of a migrant’s sense of belonging to the local communities in destination citites [4]. While ethnic self-identity starts in psychological processes of belonging and commitment, its importance and consequences are shaped by the structural contexts in which migrants live. For young rural-to- urban migrants, the destination city is not merely a new geographic setting but an institutional field marked by differentiated access to public goods, labour-market stratification, and residential segregation. These structural arrangements do not simply operate as external constraints; they are actively interpreted, internalized, and responded to through identity negotiation processes. Ethnic self-identity thus functions as a mediating lens through which migrants interpret and respond to structural conditions. This linkage between psychological identity processes and structural barriers is central to understanding how ethnic self-identity drives social integration.

The urban destinations cities where ethnic migrant youths relocate are not neutral settings but specific environments that reshape identity negotiation in distinctive ways. Five urban features are particularly important. First, the Han-dominant cultural mainstream makes ethnic minorities visibly different, keeping ethnic identity salient in ways it may not be in ethnically homogeneous home regions. Second, the differentiation in access to public services, housing, and permanent settlement, creates a legally divided urban population. Third, labor market structures channel ethnic migrants into low-skill, precarious, ethnic-niche occupations that limit both economic advancement and workplace contact with host communities. Fourth, spatial segregation in rental housing and migrant areas physically separates ethnic migrants from host communities. Fifth, the absence of established ethnic community institutions means migrants must create their own informal networks to maintain ethnic practices.

Hukou or household registration system is one such structural condition that determines many aspects of migrant’s life in cities. Although there have been a number of policy reforms regarding rural to urban migrants and the Hukou system (Household registration) in recent years [5], challenges remain as migrants are still marked as ‘*wailairen*’ or ‘outsiders’, making identity negotiation a core but under-examined mechanism. Hukou determines the livelihood and well-being of Chinese people because access to public resources and services depends on it. For young migrants from ethnic minority groups, this can be significant because geographical transition and cultural differences with mainstream urban communities may influence self-identity development, which could be a determining factor in social integration [6].

Understanding and facilitating the social integration of ethnic migrant youths is not only beneficial to the migrants themselves but also yields significant advantages for host communities and urban governance. In an ideal scenario, successful post-migration social integration would enable ethnic migrant youths to access equitable employment opportunities, public services and social networks, thereby enhancing their psychological well-being and economic contribution to the city [5]. For the host communities, integrated migrants contribute to labor-market flexibility, cultural diversity, and social innovation, while reducing inter-group tension and the social costs of segregation [4]. From a policy perspective, China and urban cities benefit from increased productivity, expanded tax bases, reduced social welfare dependency, and enhanced social cohesion. Cities that successfully integrate ethnic migrants also gain competitive advantages in attracting labor, fostering innovation, and projecting inclusive urban governance. Therefore, understanding the role of ethnic self-identity in this integration process is essential for designing effective policies that benefit all stakeholders. Ethnic self-identity could play a pivotal role in migrant’s willingness to accept the local realities and adapt to local culture which has marked differentiations from the culture of their origin. Moreover, young migrants belonging to ethnic minorities have their own language, distinct culture and lifestyle which functions as a cognitive-cultural schema while interpreting and negotiating with the adaptation process in a new environment of urban areas [7].

The existing studies mostly address self-identity of migrants as a variable to be measured and linked with other social and psychological factors rather than an experientially meaningful phenomenon that drives the adaptation and acculturation process leading to social integration. Identity is addressed as rather dynamic than static which changes based on the reference group [8]. This approach overlooks how ethnic identity is negotiated as lived, meaningful process that drives integration trajectories. As Phinney and Goosens pointed out, the geographic relocation and cultural friction between youth’s culture of origin and that of destination city influences self-identity development [9]. The specific ways of identity formation may depend on the specific cultural backgrounds and current cultural contexts of people. Their special experiences in different cultural contexts result in the specific identity development trajectories. Ethnic identity is a critical concept in the study of the so called “minority” adolescents, largely on the basis of the notion of “minority” in contrast to “majority” in mainstream societies like that of urban areas. Hence, a system oriented analysis that acknowledged the critical and ever-present role of the person’s own phenomenology or unique set of perception is paramount.

Ethnic self-identity has not received enough attention as a significant influencing factor in previous discussions about migrant’s social integration. However, ethnic self-identity plays a pivotal role in various forms of an individual’s psychological well-being including acculturation and positive social integration. As early as 1968, Erikson theorized self-identity as a phenomenon of individual exploration about his/her life positioning which unfolds within the intersecting structures of family and society and influences people’s well-being [10]. The formation of identity is a very context dependent process which requires an understanding of complex social, political, historical and cultural contexts that interacts with construction and evolution of identity [11]. The existing studies state that interactions with social, economic and political transformations influence ethnic identification but there is very limited knowledge on how it transpires among migrant youths from ethnic minorities who are suddenly exposed to the new environment of an urban area when they migrate. There is a noteworthy gap in literature about the analysis of how migrant youths from ethnic minorities integrate in the urban areas and what influences their social integration process in their migration destination. This study is different from existing research in three ways. First, while quantitative acculturation studies treat ethnic identity as a measurable predictor variable [6,12], we examine it as an experiential process. Secondly, while structural migrant integration research treats identity as an outcome of socio-economic factors [13], we treat it as a dynamic process that shapes integration. Third, we focus on the specific context of China’s rural-to-urban migration, where the Hukou system and ethnic-niche labor markets create unique structural conditions. Understanding this experiential process requires methodological approaches that attend to lived experiences and subjective meaning, positioning migrant youths as subjects of meaning rather than objects of measurement.

This study aims to analyze the social integration process of migrant youths from ethnic minorities in their destination city using their own interpretation of their experiences. A qualitative phenomenological approach used in this study relocates ethnic migrant youths from mere objects of measurement to subjects of meaning by adding contextual thickness. It sets an experientially grounded agenda for the analysis of how ethnic self-identity evolves amidst the acculturation process and its influence on social integration post-migration to urban areas. We seek to answer following research questions: 1) How does ethnic self-identity influence social integration of migrant youths from ethnic minorities after moving to cities? 2) How does the ethnic self-identity negotiate with the urban social environment post-migration to shape the new self-identity of migrant youths from the ethnic groups?

### Ethnic migrant youths among migrant population of China

China has fifty-six minority groups as recognized by the State with more than 90% of the population being ethnic Han and the rest belonging to other ethnic minority groups [14]. Ethnic minorities in China are defined mainly by four characteristics which are people of indigenous origin living in a particular territory, speaking a common language, sharing the same economy and bearing particular cultural features [15]. Most ethnic minorities come from autonomous administrative regions which is characterized as areas with more than 50% of ethnic minorities or are historically resided by indigenous minorities [15]. More than half of the poverty in the country is concentrated in these autonomous administrative regions [16]. The population of ethnic minority migrants stood at 30 million in 2014 which constituted 28% of the 106.43 million ethnic minority population [17]. The 2020 census reports state that the migrant population from the ethnic minority groups have reached 37 million which constitutes 9.8% of the total migrant population. The National Census reports state that Hui, Tujia, Miao, Zangzu (Tibetan) and Manchu ethnic minority groups are the most widely spread minority groups in China which implies that their presence has expanded across numerous provinces throughout the country [18]. Migration to cities offer the youth from ethnic minorities from these regions an escape from poverty and an attempt at securing better livelihoods in China. Ethnic migrant youths are however vulnerable in many aspects when they migrate to the urban areas including unequal employment opportunities, housing, healthcare etc. The challenges that the ethnic migrant youths face in urban areas extend beyond rural-urban disparities to include language limitations, cultural adaptation, and unfamiliar urban norms and regulations.

### Theoretical framework

Social integration of migrants in destination cities have been a topic of discussion in China since early 2000s when rural to urban migration accelerated and single person migration evolved into family unit migration [19]. Social integration of Chinese migrant population has been analyzed through various dimensions such as social, psychological, economic and demographic dimensions. The scholars who have been studying the migrant population of China have proposed various models which would aid in measuring and analyzing social integration in destination cities. However, ethnic identity has rarely been considered as an influencing factor in the study of social integration and this gap can be attributed to the fact that there is inadequate scholarly attention given to migrant population of ethnic minorities in China [18]. In an attempt to address this gap, we start by reviewing theoretical foundations related to identity construction and social integration process. We distinguish three connected constructs: ethnic self-identity, identity negotiation and social integration. Ethnic self-identity refers to how participants understand themselves as members of an ethnic group- the meaning and values attached to this membership. Identity negotiations refers to the dynamic process through which this self-identity is challenged, reaffirmed, or changed in the destination city context. Social integration refers to the multi-dimensional outcome covering economic participation, social network formation, psychological belonging and cultural adaptation.

Erikson conceptualizes identity as a subjective process of exploration, through which an individual situates themselves within the contexts of family, ethnicity and society [10]. He further posits that the status of this identity exploration is critical determinant of psychological well-being. For ethnic migrant youths the migration to urban areas is not just a change in geographic location but also a seismic shift in the very context Erikson identified as crucial. The urban areas represent a new, often dominant, mainstream culture with different norms, values and expectations where youths must navigate unfamiliar social codes which can be challenging with their rural and ethnic upbringing. The city influences their identity and the way they form identity which in turn shapes their mode and success of integration. Identity formation is a process that unfolds through the dynamic interaction between the individual and their specific environment. According to Bronfenbrenner’s Ecological Systems Theory, human development constitutes a process of perpetual adaptation, situated within and shaped by a complex, nested ecological context [20]. Ethnic social identity formation is a process of progressive, mutual adaptation between a developing person and a set of environmental systems. The impact of identity on social integration is determined by the degree of consonance and dissonance across these systems. A well balanced relationship between various systems within an individual’s ecology provides consistent, supportive messages that value the youth’s ethnic heritage, facilitates the formation of a resilient, integrated identity which forms the foundation of successful social integration.

Ethnic identity construction is a nuanced process which requires the understanding of how social, political, cultural and historical contexts interact with and influence identity formation. Phinney and Ong argue that individuals capable of integrating one’s ethnic group membership into their self-identity tend to lead healthier and more fulfilling life [21]. Migration to destination cities puts the youth in a position where new information and context about their ethnic identity is expected to challenge their existing ethnic beliefs and attitudes [22]. The consequences of constant interaction between one’s established ethnic identity and the new reality in urban destinations that involves stereotypes, cultural socialization, multiculturalism etc determines the integration of ethnic migrants into mainstream societies of the destination cities.

The four theoretical perspectives used in this study operate at different but complementary levels that together explain ethnic identity negotiation during rural-to-urban migration. Table 1 summarizes how each perspective informs a specific analytical dimension and how each dimension corresponds to the empirical themes presented in the Results. Ecological systems theory [20] provides the structural level, showing how family, community, institutions and society shape the context for integration. Eriksonian identity theory and social cognitive models [9,10] address the individual level, explaining how migrants explore and commit to ethnic identity during their transition phases. Social identity theory [23] operates at the group level, explaining how in-group and out-group categorizations shape social boundaries and integration paths. These levels work together to show how ethnic self-identity is shaped by structure, developed by individuals and expressed through group dynamics.

**Table 1:** Correspondence between theoretical perspectives, analytical dimensions, and empirical themes.

Theoretical perspective and analytical dimension	How it informs the analysis	Corresponding empirical theme(s)
Social identity theory [23], group level: in-group/out-group categorization and intergroup evaluation.	Explains how participants categorize themselves as ethnic group members, why this categorization remains salient in a Han-majority urban context, and how negative intergroup evaluations are experienced and buffered.	Primary: Ethnic self-categorization; Perceived ethnicity-based discrimination.
Eriksonian identity theory and social-cognitive models [9,10], individual level: identity exploration and commitment.	Explains how migration disrupts the contexts of identity formation and how youths re-explore, question, and recommit to ethnic identity during the urban transition.	Primary: Bicultural identity integration; Secondary: Ethnic self-categorization.
Ecological systems theory [20], structural level: nested environmental systems.	Situates identity negotiation within family, co-ethnic networks, labour-market structures, and institutional arrangements (e.g., Hukou) that condition daily interaction and integration.	Primary: Ethnic versus local language proficiency; Ethnic and cultural practices; Secondary: Perceived ethnicity-based discrimination (institutional dimension).
Acculturation framework [24], outcome level: acculturation positions.	Provides the typology (integration, segregation, assimilation, marginalization) used as an interpretative lens for the overall pattern of integration outcomes.	Primary: Bicultural identity integration; interpretative lens for the cross-theme pattern of strong identity retention with limited host-community contact.

Table 1 clarifies how the four theoretical perspectives jointly informed our analysis. The five empirical themes were not derived deductively from these theories. Consistent with the inductive commitments of IPA, the themes emerged from exploratory comments that were clustered into emergent and super-ordinate themes on a case-by-case basis, as described in the Methodology. The theoretical perspectives were engaged at the interpretative stage: social identity theory illuminates the group-level processes underlying ethnic self-categorization and perceived discrimination; Eriksonian identity theory explains the individual-level renegotiation that culminates in bicultural identity integration; ecological systems theory situates language proficiency and cultural practices within nested structural contexts; and Berry’s framework provides the outcome typology through which the overall pattern of strong identity retention with limited host-community contact is interpreted. Several themes are informed by more than one perspective, reflecting the multi-level nature of identity negotiation in structurally segregated migration settings

The scholars in China have identified four types of social integration among migrants based on socio-economic indicators and cultural psychological indicators namely integration, segregation, assimilation and marginalization [13,18]. The most common factor determining the type of social integration is the level of cultural identity that the migrants retain or maintain while trying to adapt to their destination cities. In the integration type of social integration, migrants from ethnic minorities retain their ethnic and cultural identity at the individual level while they interact with members of host communities without much differentiation. The ethnic migrants are well-accepted by local host community members but cultural boundaries remain. The segregation type of social integration refers to the condition of ethnic migrants where they strongly guard their ethnic identity while avoiding contact with local communities and also being excluded in community and social activities. Marginalization pertains to the situation where ethnic migrants have minimal possibility or desire to maintain their ethnic identity but are also excluded from host communities. The fourth type is assimilation which occurs when ethnic migrants do not intend to maintain their ethnic identity and actively seek to interact with host communities to be accepted as a part of them.

The existing theories offers a foundation for examining how identity is shaped upon migrating to urban areas but it does not clarify how ethnic identity or migrants belonging to ethnic minority groups impact social integration in destination cities. This study will focus on how ethnic self-identity negotiates with the new realities faced by ethnic migrants and influence their social integration in destination cities.

## Methodology

This study follows a qualitative design using two analytical components, each with a distinct function. First, Interpretative Phenomenological Analysis (IPA) is the overall methodological framework. It guides the study’s approach to data collection, analysis, and interpretation. Second, thematic analysis procedures [25] serve as the cross-case patterning method within IPA. They organize individual case analyses into themes that allow comparison across participants. This two-part design ensures that IPA remains the overarching framework while allowing systematic cross-case comparison and theoretically informed interpretation.

All ethnic group names, methodological terms, and key theoretical concepts follow the standardized usage specified in Table 2, and the full manuscript, including participant attributions beneath interview excerpts, was checked against this table. Participant codes and demographic attributions were additionally verified against the master coding sheet.

**Table 2:** Standardized terminology used in this study.

Term	Standardized usage	Definition/ scope
Miao	“Miao” (not “Miao Zu” or “Miaozu”)	One of the most widely distributed ethnic minority groups in China [18]; participants originated from Guizhou and Guangxi
Tujia	“Tujia” (not “Tujia Zu” or “Tujiazu”)	One of the most widely distributed ethnic minority groups in China; participants originated from Hunan
Zangzu (Tibetan)	“Zangzu (Tibetan)” at first mention; “Zangzu” thereafter	One of the most widely distributed ethnic minority groups in China; participants originated from the Xizang (Tibet) Autonomous Region
Hui	“Hui”	The researcher’s own ethnic group; not a participant group in this study
Han	“Han”	The ethnic majority group in China; local residents and institutions are referred to as the “host community”
Interpretative Phenomenological Analysis (IPA)	Full term, capitalized, at first mention; “IPA” thereafter	A qualitative approach exploring how individuals experience and make sense of significant life experiences [26].
Ethnic self-identity	Hyphenated; “ethnic self-identity”	An individual’s subjective sense of belonging to an ethnic group, incorporating commitment, belongingness, and the value attached to membership
Ethnic self-categorization	Hyphenated; “ethnic self-categorization”	The act of labelling oneself as a member of an ethnic group
Social integration	“Post-migration social integration” at first mention; “social integration” thereafter	The multi-dimensional process through which migrants establish social, economic, and psychological ties within the destination city
Destination city	“Destination city” (replacing “urban destination” and “host city”)	The city to which participants migrated; in this study, Wuhan, Hubei Province
Host community	“Host community” (replacing “host society”)	Local, predominantly Han residents and institutions of the destination city
Ethnic migrant youths	“Ethnic migrant youths” (replacing “young ethnic migrants” and “ethnic minority migrant youth”)	Persons aged 15–29 from ethnic minority groups who migrated from rural areas to cities; all interviewed participants were aged 18–26
Hukou	“Hukou”, capitalized, glossed as “household registration” at first mention	China’s household registration system, which conditions access to urban public services
‘Earn and return’	In single quotation marks at first mention; without quotation marks thereafter	Participants’ expressed orientation of working in the city temporarily and returning to the home region
Emergent theme	“Emergent theme” (not “emerging theme”)	A theme arising from exploratory comments during case-level IPA analysis
Super-ordinate theme	Hyphenated; “super-ordinate theme”	A higher-level theme clustering related emergent themes across cases

Interpretative Phenomenological Analysis (IPA) helps to understand how ethnic identity impacts the social integration of the ethnic migrant youths. The participant’s experiences of migration and subsequent process of integrating into the urban communities is treated as a subjective matter. Introduced by [26] in the UK, IPA has gained a global prominence as an integrative approach that synthesizes established philosophical traditions such as phenomenology. IPA is phenomenological in its commitment to exploring individual’s lived experiences and their personal perception of phenomena. IPA is one of the most suitable approaches when a study requires epistemic and procedural core designed to understand how ethnic- migrant youths themselves interpret, experience and give meaning to their ethnic self-identity while relocating from rural ethnic enclaves to cities with diverse population. We aim to analyze the impact of ethnic identity on social integration of ethnic youth from their lived experiences and their perspectives rather than limiting them in the frame of ‘one size fits all’ approach by using established questionnaires to measure their ‘degree of integration’. Phenomenology in this context accesses the ‘lived’ experience of being a Zangzu, Miao and Tujia person. Furthermore, IPA follows a double hermeneutic approach which implies that participants make sense of their personal and social world and the researcher tries to assign meaning to the participant’s sense of experience [27]. Hermeneutics allows the researcher to make sense of those lived experiences in relation to the social and power context of migration.

We selected IPA because it fits with the nature of our research questions. IPA focuses on understanding how people experience and make sense of their world [26,28]. This is appropriate for our study for three reasons. First, ethnic identity is subjective and personal, requiring exploration of how participants perceive their ethnic belonging rather than external measurements. Second, the researcher must interpret participant’s interpretations because ethnic identity stories are embedded in cultural and linguistic frameworks that surface level descriptions cannot capture. Third, in-depth individual analysis is important because identity negotiation varies across individuals and ethnic groups, making broad generalizations potentially misleading.

**Fig 1:**
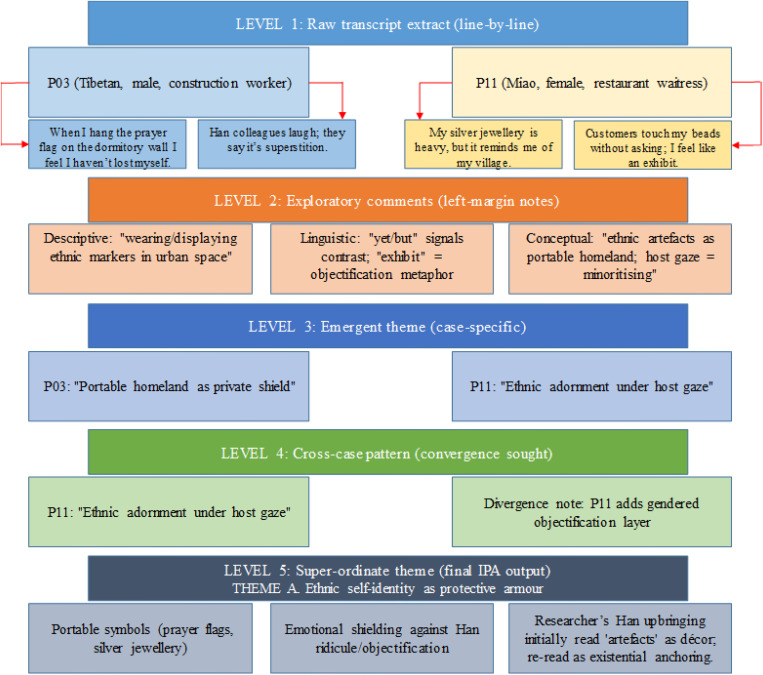
IPA Coding Tree: From Exploratory Comments to Super-ordinate Themes.

To illustrate the analytical process of IPA and demonstrate how we moved from raw data to theoretical insights, Fig 1 presents the coding tree that emerged from our analysis of two participant cases. This visual representation exemplifies the five-level analytical progression that characterized our interpretative phenomenological approach.

IPA’s double hermeneutics captures both personal significance and its social resonance. In this study, we focus on ethnic migrant youth’s lived experiences regarding their attempts and efforts at integrating in mainstream society and communities in destination city and how they assign meaning to those experience with ethnic identity as a backdrop of that meaning making. Furthermore, symbolic interactionism or exploring the participant’s inside view on the phenomenon being analyzed is central to IPA. We constantly analyze the meaning that migrant youths ascribe to events in their life as they adapt and integrate in cities and explore how those meaning are obtained through a process of social engagement and interpretation [28]. The researcher belongs to Hui ethnic minority of China. The researcher had predisposed knowledge and first-hand experience of belonging to an ethnic minority in China and the intricacies that ensued. This positionality brings both benefits and challenges to the research. Benefits include cultural and linguistic familiarity with Mandarin Chinese ethnic discourse, which helped build rapport; experiential understanding of minority-majority dynamics, which sensitized the researcher to participants’ accounts of discrimination; and shared minority status, which may have encouraged disclosure. Challenges include potential over-identification with participants’ stories, which could reduce critical distance; differences between the researcher’s Hui identity and participants’ Miao, Tujia, and Zangzu identities, which may have created interpretative gaps; and the researcher’s urban educational background, which may have introduced class-based differences in interviews. To address these challenges, author requested research team-mates and advisors from different backgrounds to review interpretative claims, and the first author maintained a record of moments when strong emotional reactions to participants’ stories occurred during analysis.

Semi-structured questions were designed to collect data through interviews. The collected data was transcribed and translated into English for interpretative analysis, with cross-case patterning organized using thematic procedures. Thematic analysis supports the analysis of data within a specific context given the meanings that an individual attribute to them which makes this method compatible with IPA. Aligned with the theoretical foundations of IPA, this study explores ethnic migrant youths’ lived experiences of social integration in destination cities, with particular attention to the role of ethnic identity. The data for this study was collected from 11^th^ January 2025–27^th^ July 2025 in Hubei province. The researchers first collected information on communities where migrant population mostly live. Then they approached the social workers and community service centers of those communities to get in touch with the ethnic migrant youths. After initial meeting with the ethnic migrant youths, the researchers requested them if they could help contact more members of their ethnic groups in Hubei who would be willing to participate in the interview. Hence, a snowball approach of sampling was used for this study.

The research question consisted of questions regarding ethnic identity and social integration. The interviews started with general questions about demographic details which moved on to questions about their ethnicity and its significance to them as a migrant who decided to move to urban areas. The questions included their opinion on what it means to belong to their particular ethnic group. Furthermore, researchers asked the participants if their ethnic identity has impacted their experience of being a migrant in the cities and how. This then led on to the question of how their belonging to a certain ethnicity has influenced integration process in the destination city and/or if at all. The interview then focused on their idea of integrating into the destination communities and how their ethnicity has impacted the process and what is helpful or unhelpful in the integration process.

Each interview was digitally recorded and was transcribed verbatim then translated into English by the researcher and cross checked by a bilingual researcher whose native language was English but was proficient in Chinese. Translation into English followed a three-stage procedure designed to minimize distortion. Stage 1: The first author (Mandarin native speaker, fluent in English) produced initial translations, attending to both literal meaning and cultural and emotional content. Stage 2: A bilingual researcher (native English speaker, proficient in Mandarin) reviewed all translations for accuracy and nuance, flagging culturally specific terms for discussion. Stage 3: The research team met to resolve discrepancies and document decisions about key terms in a record. For concepts with no direct English equivalent, we kept the Mandarin term with an explanatory note to preserve cultural specificity. The researcher read every transcript twice while listening to the audio and entering exploratory comments in the left margin of a three-column IPA worksheet as descriptive (what happened), linguistic (metaphors, gestures) and conceptual (initial abstractions). The analytical process followed IPA’s recommended steps from individual case analysis to cross-case combination, with clear criteria at each stage. For initial coding, we recorded three types of exploratory comments: descriptive (what happened), linguistic (metaphor, emphasis, gesture), and conceptual (initial abstractions), following Smith et al. [29]. For theme development, a comment cluster was elevated to a theme only when it met three criteria: (a) it appeared in multiple transcripts within at least two ethnic groups; (b) it captured something meaningful about how participants experienced the relationship between identity and integration; and (c) it was expressed with emotional intensity. For main theme construction, we examined themes for logical relationships, grouping sub-themes under main headings when they shared a common function. Unusual cases, participants whose accounts differed from dominant patterns, were noted and analyzed for the limits they placed on our interpretations. The researcher requested other researchers to discuss the themes and moved iteratively between individual themes and the entire data set following IPA’s ‘zoom-in/zoom-out’ principle. Divergent cases were deliberately scrutinized to ensure that minority voices were not flattened. The preliminary themes were grouped into five super-ordinate themes. Extracts were selected that best illustrated each theme’s essence and its internal variations.

In IPA, credibility is established through transparency, reflexivity, case depth, and interpretative plausibility [30,31]. We ensured transparency by documenting all analytical stages from exploratory comments to main themes. We practiced reflexivity by examining how the first author’s positionality shaped interpretation. We achieved case depth through detailed individual case analysis before cross-case comparison. We established interpretative plausibility by aligning our findings with existing literature on acculturation, social identity, and migrant integration.

Informed consent was collected from all the participants in written. The contents of the form were also read by the researchers before each interview to ensure that the participants understood significant points such as participation being voluntary, no compulsion in answering any question they are not willing to, their right to leave the interview at any point and decide what to do with the information already provided. The participants were informed that that their responses would be anonymized and published in research data and no identifying details would ever be published or disclosed anywhere. To anonymize the identity of the participants, a code was assigned to each participant while storing the data. The majority of the participants belonged to middle or lower socio-economic backgrounds with a range of educational backgrounds but all below high school. The study was approved by the Ethical review committee of Ningxia Vocational and Technical University (Ningxia Open University). This research represents an independent, unfunded qualitative inquiry conducted by the first author.

### Participants

National census statistics indicate that the Hui, Tujia, Miao, Zangzu, and Manchu are among the most widely distributed minority groups in China, meaning that they are distributed across various provinces throughout the country [18]. The researchers were able to contact participants from Miao, Tujia and Zangzu ethnic groups for this study. They all had the common background of belonging to an ethnic group, having an ethnic language, having migrated to urban area at least three years before, living in the same community in migration destination and similar age bracket. A total of 30 participants were interviewed. A small sample size is acceptable when using IPA as it is an idiographic method focused on depth [32]. Smith and colleagues [29] have stated that 4–10 participants are the best number of samples in phenomenological research but also mention that ‘no right answer exists to the question of an adequate sample size’ and it would depend on nature of the research problem and potential yield of the findings [33]. The larger sample reflects a practical decision: the study’s research questions require cross-ethnic comparison, which needs sufficient representation in each of three ethnic groups. We managed the tension between individual case depth and cross-group breadth through a two-phase approach. In Phase 1, each transcript was analyzed case-by-case using the IPA three-column worksheet (descriptive, linguistic, and conceptual comments) to develop themes specific to each participant. In Phase 2, these case-level themes were compared across the sample to construct main themes. This approach preserves individual case depth while enabling systematic cross-group comparison. We acknowledge that this design trades some case depth for cross-ethnic breadth, which is a limitation of this study. The demographic details of participants are presented in Table 3 below:

**Table 3:** Demographic Characteristics of Participants by Ethnic Group (N = 30).

Ethnicity	Number of participants	Sex	Age	Educational level	Time Lived in City (in Years)	Occupation
Miao	9	4 Male; 5 Female	18-24 years	High-School Drop Out (3 Female, 1 Male), Middle School Passed (2 Female, 1 Male) Primary School completed (2 Male)	3-6	Street Vendor (2), Food Service Worker (1), Retail Street Vendor (2), Construction workers (4)
Tujia	10	4 Male; 6 Female	20-26 years	High-School Completed (2 Male) High School Drop Out (2 Female 1 Male) Middle School Completed (1 Female) Vocational School Graduated (3 Female, 1 Male)	3-7	Street Vendors (2),Retail Service Assistant (1), Construction Workers (4), Retail Assistant (2), Building maintenance worker (1)
Zangzu	11	6 Male; 5 Female	19-25 years	High School Graduated (2 Male 2 Female) High School Drop Out (1 Female) Middle School Completed (2 Male) Vocational School completed (2 Female, 2 Male)	3-4	Street Vendor (3), Personal Service Worker (1), Food Service Worker (1) Sanitation Worker (2) Construction worker (4)

The educational profile of the participants reflects the structural educational differences in ethnic minority regions [17]. All participants had completed no more than high school, and several participants only had primary or middle school education. This limited education directly shaped their employment paths, leading most into low-skilled, informal-sector jobs such as street vending, construction, and restaurant service. These jobs resulted in poor housing conditions in migrant areas. Limited education also constrains their ability to learn Mandarin and to navigate urban administrative systems. This shows that educational background compounds the barriers to social integration beyond ethnic identity alone.

Regarding migration circumstances, most participants had migrated alone to seek employment, while some had relocated with spouses and family members. Most participants maintained regular contact with relatives and friends in their home communities through mobile phone and internet connectivity. Many reported that fellow villagers or relatives had also migrated to Wuhan, forming ethnic networks in the destination city. The geographical distances between participant’s home areas and Wuhan were substantial: Hunan province is approximately 350–500 km from Hubei, while Guizhou and Guangxi are roughly 800–1000 kilometers away, and the Xizang (Tibetan) Autonomous Region is over 2000 kilometers from Wuhan [34]. These distances reinforced the ‘earn and return’ orientation among participants, as permanent settlement would mean severing ties with home communities.

Some interviews were conducted at the community service center between researcher and the participant while others were conducted at participant’s choice of place which was usually in their homes. The interviews lasted between 55–90 minutes. Most participants were interviewed once to thrice which gave researchers an opportunity to delve deeper into participant’s experiences and meaning making process. The interviews were conducted in Mandarin Chinese to maintain the uniformity across language. All the participants had their ethnic language and had varying levels of Mandarin Chinese which was deemed sufficient for the purpose of this interview.

## Results and findings

Cross-case patterning was organized using an Excel matrix to facilitate systematic comparison across participants, while remaining within the IPA framework. The thematic analysis procedure served as the cross-case patterning method within IPA which organized individual case analysis into themes that allowed comparison across participants. The collected data was transcribed verbatim in Mandarin Chinese then translated into English for coding and identifying themes. The researcher read all the interview multiple times. The participants were contacted again for a second and third interview if the researcher identified themes that could be explored further for a deeper understanding of the participant’s ethnic identity and social integration processes. To demonstrate sustained case depth, we include detailed case illustrations for one participant from each ethnic group (e.g., P11 from Miao, P22 from Tujia, P09 from Zangzu), showing the full analytical trajectory from descriptive comments through linguistic and conceptual analysis to emergent themes. These case illustrations demonstrate that individual depth was not sacrificed for breadth. The cross-case patterning was organized using thematic procedures within the IPA framework, not as a separate methodology

The results show that there are five major factors of ethnic identity which impacts social integration in destination city for ethnic migrant youths. Ethnic self-categorization, Language proficiency, Cultural practices, perceived ethnicity based discrimination and bicultural identity integration are the five factors of ethnic identity which impact social integration of ethnic migrant youths in destination cities. We also noted a gender difference in participant’s response to host communities’ behavior towards migrants and its subsequent impact on social integration which is detailed in a subsection in results and findings section. Five factors presented below are not independent factors that affect social integration separately. Rather, they represent connected experience strands through which ethnic self-identity interacts with the urban context to shape integration paths. Each factor captures a different aspect of this interaction: self-categorization and cultural practices express identity; language proficiency and perceived discrimination represent contextual conditions filtered through identity; and bicultural integration represents a negotiated outcome. Together, they show a two-sided pattern: ethnic self-identity both protects migrants against urban marginalization and marks social boundaries that complicate host-community integration.

### Ethnic self- categorization

Ethnic self-categorization in this study refers to migrant youth’s act of referring oneself as ‘Zangzu’, ‘Tujiazu’ (Tujia Ethnic group) or ‘Miaozu’ (Miao ethnic group). Although it may appear to be a simple act of labelling oneself, ethnic self-categorization functions as a cognitive anchor that shapes how ethnic migrant youths interpret, navigate, and ultimately integrate into destination-city communities. The migrant youths from all three ethnic groups had similar opinion about ethnic self-categorization. Defensive self-labelling as dignity preservation was noticed across migrants of all ethnic groups which also served as a coping mechanism.

*‘No matter where I go in the country, the first and foremost it is important for me to be who I am. I belong to Zangzu ethnic group. When I interact with other people, I interact as a Zang Zu person. I wear this ethnic identity proudly. I know people in cities might think of me differently if they knew I come from Xizang (Tibet) but I still want them to know when they interact with me*.’ P1, Male, Zangzu Ethnic Group.

This account exemplifies defensive self-labelling as dignity preservation. The participant does not merely report his ethnicity; he announces it pre-emptively (‘it is important for me that they know me as a person belonging to Zang Zu ethnic group’) before any interaction has occurred. The clothing metaphor ‘I wear this ethnic identity proudly’ frames identity as something deliberately displayed rather than passively held, while the contrastive construction ‘people in cities might think of me differently… but I still want them to know’ signals that assertion occurs in full awareness of anticipated stigma. Self-categorization here operates as anticipatory coping: by claiming the category before others can impose it, the participant retains authorship of its meaning. This pattern of pre-emptive, dignity-preserving self-labelling recurred across participants from all three ethnic groups.

A secure ethnic self-categorization develops confidence when interacting with others in the explorative environment of a destination city. It facilitates ethnic migrant youth’s ‘approach rather than avoidance’ behavior in the destination communities. The stereotype awareness supports reactive identity assertion.

‘*There are many stereotypes and biases against us in cities because we come from rural areas and belong to a different ethnic group. Some think our hometowns are so rural and we are so poor because we are lazy or we don’t work hard enough. We are poor in our hometowns because we do not have economic opportunities like in cities and not because we are not willing to work hard. Being a person of Tujia is almost synonymous to being poor. But it does not affect who I am.’* P11, Male, Miao Ethnic Group.

In the post migration context, urban exposure allowed reflexive ethnic consciousness in many participants. The exposure to mainstream society and communities of urban areas gave ethnic migrant youths a broader perspective on how they are viewed outside of their hometowns. It gave them an opportunity to analyze their position in their destination cities and host communities.

‘I was 17 w*hen I left my hometown for the first time and came to a city. I did not know* *much about outside world because back in my hometown we all speak the same language, eat same food, do same kind of work for living. So I was surprised to learn that I was perceived as an outsider not only meaning that I came from another place but that I am ‘different person’ meaning I come from a different ethnicity. I did not know that for city people it is such a big deal. I thought we all belonged to our country and were same. At first it made me feel a bit shameful but later I asked myself why should I feel shame just because I do not fit among city people. I can’t change where I was born.’* P10, Male, Zangzu Ethnic Group.

This excerpt captures the reflexive ethnic consciousness produced by urban exposure. The narrative moves through three stages: unawareness (‘I thought we all belonged to our country and were same’), imposed categorization (being perceived as a ‘different person’), and reflexive reappraisal, marked by the rhetorical question ‘why should I feel shame just because I do not fit among city people’. The shift from feeling ‘a bit shameful’ to the declarative ‘I can’t change where I was born’ reveals the participant converting an externally imposed, stigmatized category into an accepted fact of self. Analytically, the excerpt demonstrates that ethnic self-categorization is not imported unchanged from the place of origin; it is constituted in the city, where the Han-majority context forces category salience. This trajectory from imposed difference to reflexive acceptance was characteristic of participants who migrated in late adolescence.

The participants often mentioned that in their hometown there was no need to categorize themselves as ‘ethnic’ because everyone shared the same language and practices but migrating to city forced the level ‘different ethnicity’ or ‘outsider’ into their consciousness. This situation forced them to negotiate their existing ethnic self-identity with new reality of being a migrant, being an outsider in a city and belonging to a non-local ethnic group. It is evident in the data that these factors influenced their decision on whether or not to communicate with local community or put effort into interacting with members of host communities to decrease the social distance between them.

### Ethnic vs local language proficiency

Ethnic language was one of the most distinct components of ethnic identity that set the ethnic young migrants apart from others in the destination cities might it be other migrants or locals. Young migrants from all ethnic groups experienced constant friction between upholding one’s ethnic language as opposed to gaining proficiency in local language which is Mandarin Chinese. Mandarin Chinese is the administrative Lingua Franca in China. Having good proficiency in Mandarin Chinese or local dialects brings the benefit of not just better job opportunities, school placements in public schools, any administrative work in government offices etc, it also increases trust from the local residents which facilitates the social integration process.

‘*When I was looking for work in cities, my elders who have longer experience of working there told me that I should try to speak Mandarin and try to learn more because it makes it easier to find work. The boss for whom I worked for the first time said I am a hard worker because I have learned Mandarin well. It felt strange because before that I never assumed that speaking a different language could be associated with how hard a person works. As much as I want to improve in speaking Mandarin sometimes I do not want to talk to new people.’* P6, Male, Tujia Ethnic Group.

The excerpt reveals how Mandarin proficiency is moralized in the urban labor market: the employer reads language acquisition as evidence of diligence (‘I am a hard worker because I have learned Mandarin well’). The participant’s marker of dissonance, ‘It felt strange’, registers his recognition that a linguistic attribute is being treated as a character judgement. Most analytically significant is the closing contrastive: ‘As much as I want to improve in speaking Mandarin sometimes I do not want to talk to new people.’ In a single sentence the participant articulates the approach–avoidance conflict at the heart of the protective-boundary mechanism: the instrumental pull toward the host language coexists with withdrawal from the very interactions through which proficiency would develop. This tension between linguistic investment and interactional retreat appeared across all three ethnic groups.

The influence of ethnic identity on securing employment emerged as an important finding in the data. Participants reported that ethnic identity shaped their employment opportunities in several ways. First, Mandarin proficiency directly affected hiring prospects and workplace evaluations. P6 recounted how his boss associated his Mandarin learning with work ethic: *The boss for whom I worked for the first time said I am a hard worker because I have learned Mandarin well.* Second, ethnic discrimination in hiring was reported by several participants. P11 stated: *As an ethnic minority person, I have faced discrimination getting jobs, the bosses think it is hard to communicate with us because of our accent and don’t want to take the trouble.* The lack of proficiency desired in the urban areas also acted as a source of discrimination against the ethnic migrant youths. The influence of the ethnic language or dialect when speaking Mandarin Chinese would give away ethnic migrant’s identity as not belonging to mainstream urban community or society.

*‘People in cities mock how we speak Mandarin Chinese. They have absolutely no understanding that it is not our mother tongue and we did not grow up speaking that language in our day to day life. I speak as less as possible to local people or actually with anyone who doesn’t understand my ethnic language.’* P5, Female, Miao Ethnic Group.

This account traces the pathway from linguistic mockery to social withdrawal. The participant first establishes effort and legitimacy (‘We put effort into learning Mandarin as much as we could’) before describing her response: ‘I speak as less as possible to local people or actually with anyone who doesn’t understand my ethnic language.’ The escalation from ‘local people’ to ‘anyone’ marks a generalization of avoidance beyond the original source of mockery, converting a specific injury into a standing interactional strategy. The excerpt provides direct experiential evidence for the reverse pathway proposed in the Discussion: anticipated ridicule motivates identity-protective withdrawal, which reduces contact opportunities and forecloses the very conditions under which prejudice might otherwise be reduced.

However, ethnic language use is directly related to ethnic self-categorization and ethnic language use was found to act as buffer against discrimination stress. Most of the participants liked to keep a close contact with members of their own ethnic groups who had also migrated from their place of origin. Many participants expressed that they feel a strong sense of belonging which provides them comfort when being together with people from their own ethnic group in their destination cities. Intra-ethnic social networking is an important way for participants to maintain ethnic identity and cope with urban stress. Participants described several ways they maintained contact with same-ethnic members in the destination city. Most commonly, they used WeChat groups organized by home village or ethnic affiliation to share information about employment and housing, participants described getting together with fellow villagers and townsmen during holidays. These networks are informal and organic rather than formal associations.

‘*I always get comments about the way I speak Mandarin. Some people make me feel inferior about it. I started admiring my ethnic mother tongue after I migrated to city and realized how precious it is that I can speak it. Sometimes during holidays, we get together with my fellow villagers and townsmen and speak our own language without anyone judging us. I feel kind of freedom in those times. It is probably the freedom of not being constantly judged for speaking* *differently or for being an outsider*.’ P4, Male, Zang Zu Ethnic Group.

Language functions as a strong component of ethnic self-identity. Most participants in this study favor retaining their ethnic language as the expense of better opportunities that Mandarin proficiency might bring. The language proficiency functions as a tool of integration and source of conflicting emotions for ethnic migrant youth in cities.

### Ethnic and cultural practices

As the ethnic migrants moved away from their hometowns, the distance from their ethnically and culturally rich environments increased but they also received exposure to mainstream cultures of the urban area. They faced difficulty in maintaining day to day cultural practices of their ethnicity in an urban area but continuing the feasible ones maintained their sense of connection to their ethnicity and hometown. This had a significant impact on their sense of belonging both to their place of origin and destination cities.

*‘Our daily routine is determined by our work which is pretty much the same every day. Back home we have festivals to celebrate important occasions. We even celebrated festival to taste new rice. But here we don’t even know when those festivals come and pass. I don’t think I want to stay all my life not enjoying our local festivals. They really rejuvenated us from daily life stress.’* P3, Male, Tujia Ethnic Group.

Most of the participants expressed dissatisfaction at not being valued for their ethnic heritage in the cities. The mainstream society’s disregard for ethnic minority groups and their ethnic heritage was difficult to accept for most of the participants who came from ethnic enclaves where ethnic and cultural practices formed a core part of daily life.

*‘Without our ethnic cultural practices in our daily life, I feel very invisible in the city. It is as if no one sees me or have any regard for who I am. Back in my hometown we regularly visited monasteries to light an incense or pray. It gave me a sense of peace during difficult times. So I sometimes feel out of place living in the city.’* P9, Female, Zangzu Ethnic Group.

The metaphor of invisibility (‘I feel very invisible in the city. It is as if no one sees me’) connects the absence of cultural practice space to a felt loss of personhood. For this participant, religious practice was not a discrete activity but an emotional-regulation resource (‘It gave me a sense of peace during difficult times’), whose disruption produces ‘placelessness’ (‘I sometimes feel out of place living in the city’). The excerpt reveals that cultural practice discontinuity undermines belonging through two channels simultaneously: the loss of a coping resource and the loss of recognition, in which the city’s indifference to ethnic heritage is experienced as indifference to the self. Among Zangzu participants in particular, religious practice emerged as the most acutely missed dimension of cultural continuity.

For some participants, ethnic art and practices formed an integral part of their life which was discontinued as they migrated to cities. It was not just a missing part of their normal life from the place of their origin but also a disruption from a familiar way of life. This further enhanced their feeling of being disconnected from the surrounding they were in the cities.

‘*Back home our lifestyle is filled with art and crafts. Tujia people weave and embroider a lot. All our dresses and many things we use in our daily lives are colorful and vibrant. My grandmother and mother taught me how to create art. Although cities are filled with colors, I do not feel connected to those colors and art here. Sometimes when we meet in the park, we try to dance. I am scared of forgetting the steps. When I miss all of that about my village I feel so out of place here in the city*.’ P17, Female, Tujia Ethnic Group.

This excerpt illustrates embodied cultural transmission and its anticipated rupture. Craft and dance are described as inherited through the female line (‘My grandmother and mother taught me how to create art’), making practice continuity inseparable from family identity. The participant’s fear, ‘I am scared of forgetting the steps’, expresses anticipated identity loss in bodily terms: what is at risk is not knowledge about the culture but the capacity to perform it. The contrast between the city’s abundant but alien colors (‘cities are filled with colors, I do not feel connected to those colors’) and the meaningful colours of Tujia craft renders cultural distance in sensory language, showing that disconnection is experienced aesthetically as well as socially. Informal re-enactments, such as dancing with co-ethnic friends in parks, function as partial repair work for this rupture.

The cultural distance between place of origin and destination city emerged as one of the determining factors of social integration. The lack of opportunity and space for cultural practices in city had negative impact on migrant’s sense of belonging to the cities. It further drew them closer to their own townsmen than members of local host communities. Participants did not report having formal ethnic associations or organizations. Instead, they relied on natural connections based on shared origin, family ties and word-of-mouth referrals. These networks provide emotional support and practical help but also reinforce ethnic boundaries that limit integration with host communities.

### Perceived ethnicity based discrimination

Ethnicity based discrimination was a common experience across all the participants from all ethnic groups. Ethnic migrants face twice more discrimination as they are first regarded as ‘outsiders’ implying that they do not have household registration in the city and secondly ‘outsiders’ in the sense that they do not belong to the majority ethnic population of the city which is most often Han ethnic group. The participants report experiencing discrimination for belonging to an ethnic minority group as Han mainstream society perceive them as outgroup.

‘*As an ethnic minority person, I have faced discrimination in getting jobs. The bosses think of us as being lazy. If we were lazy would we leave our hometowns and come so far to the cities to find work? People here do not realize that we have made twice as much effort. It really makes me feel angry*’ P14, Male, Miao Ethnic Group.

The excerpt presents the ‘lazy’ stereotype and its experiential refutation. The participant’s rhetorical question, ‘If we were lazy would we leave our hometowns and come so far to the cities to find work?’, inverts the stereotype by citing migration itself as evidence of industriousness, while ‘twice as much effort’ quantifies the double burden carried by ethnic migrants, who must overcome both outsider status and ethnic prejudice. The closing statement of anger (‘It really makes me feel angry with city people’) is analytically important: unlike the shame described elsewhere in the data, anger is an other-directed emotion that preserves self-worth while widening social distance. The excerpt thus reveals, within a single account, how perceived discrimination simultaneously protects dignity and reinforces the boundary that complicates integration.

Participants reported facing both covert and overt forms of discriminations. Some forms of discrimination were not intended but is so embedded in the mindsets of the mainstream society that it is not realized as a form of bias or discrimination. Such acts of discrimination further encourage the migrants to maintain a distance with members of the host communities.

*‘I look visibly different as an ethnic minority person. So, in busses and metro a lot of people do not like to sit beside me. It feels somehow hurtful when I sit in the available seat in bus or metro* *but the person next to me will move to another seat or just stand after I sit beside him or her. It feels like discrimination based on how I look. It feels strange because this person does not even know who I am but somehow feels uncomfortable or scared to sit beside me.*’ P22, Female, Zangzu ethnic Group.

This account exemplifies covert, visibility-based discrimination. The participant is excluded not through words but through movement (‘the person next to me will move to another seat or just stand’), and her observation that ‘this person does not even know who I am’ identifies the depersonalizing core of the experience: she is responded to as a category, not as a person. The hedged formulation ‘It feels like discrimination based on how I look’ reveals the interpretative labour demanded by covert exclusion, in which the target must infer intent from behaviour that is never made explicit. Such micro-level exclusions in anonymous public space were reported across the sample and were described as more corrosive to belonging than overt insults, precisely because they admit no possibility of response or correction.

One of the themes that emerged recurrently in the data was how lack of information or misinformation about ethnic minority groups helps in formation of prejudice or acts of discrimination against minority groups. The participants expressed dissatisfaction towards how majority of the population do not have enough knowledge about lifestyles of ethnic minority groups but form judgements based on limited knowledge or outdated information that is not applicable anymore.

‘*By now it is normal for me to be judged by people in cities as being poor because I belong to ethnic minority. What astonishes me is that they think we are all dirty and lack hygiene in daily life. Maybe that was the condition in our villages fifty or hundred years before but things have changed. I find it strange how deeply such misconceptions exist in people of the cities who think* *of themselves as superior to everyone else.’* P16, Female, Tujia Ethnic Group.

Perceived ethnicity based discrimination has by far emerged as the strongest psychological barrier to social integration among ethnic migrant youths in this study. Ethnic migrant youths who reported being exposed to ethnic-accent mockery or ‘uncivilized’ stereotypes expressed low desire to make efforts to interact with members of local communities. Perceived ethnicity based discrimination was also mentioned as one of the primary reasons why ethnic migrant youth choose to return to their hometowns ultimately. These factors related to perceived ethnicity based discrimination severely undermine ethnic migrant youth’s social integration process in destinations cities.

### Bicultural-identity integration

Bi-cultural identity integration refers to the degree to which a person experiences their ethnic and mainstream cultural identities as compatible or conflicting. Although ethnic migrants had more of conflicting experience regarding their ethnic identity and identity they developed in destination cities as migrants, they still felt affinity towards the destination city. To some degree this contributes to the process of social integration.

*‘I have decided that I will ultimately go back to my hometown but I also respect this city for providing me with an opportunity to earn and afford a better living standard for my family. Although I don’t call it my home or even second home, I have started feeling comfortable being here. I will probably always be a migrant to this city but even when I leave from here I might want to come back every now and then to feel that I once belonged to this city.’* P24, Female, Miao Ethnic Group.

This account expresses attachment without membership. The participant disclaims the strongest forms of belonging (‘I don’t call it my home or even second home’) while reporting growing comfort and an imagined future return ‘to feel that I once belonged to this city’. The paradoxical formulation ‘I will probably always be a migrant to this city but… I might want to come back’ presents bicultural integration as a partial, retrospective, and affective bond rather than a settlement decision. The gratitude expressed toward the city (‘I also respect this city for providing me with an opportunity’) indicates that, even within an earn-and-return orientation, the urban experience is incorporated into the self-narrative. This supports our argument that acculturation positions are fluid and situationally contingent rather than fixed categorical outcomes.

Bicultural identity integration also acts as a coping mechanism for migrants against the discrimination they faced. Although they are perceived as ‘outsiders’ ethnic migrants gradually accept the formation of their new identity which often is a blend of their existing identity as belonging to an ethnic group from the place of their origin and belonging to the city of their choice at least temporarily.

‘*I do not belong to this city. But now that I live here I feel like I am also a part of this place. I do not have local friends or relatives here but I feel like I am somehow related to people here. Once I brought my family member to city for treatment during a major festival holiday and most restaurants were closed. The family of the person next to my wife’s bed gave us food daily for many days. The person said we are locals here, we have a home and we can manage food during holidays It felt like I suddenly found a relative in this city. For the first time I met someone who did not see me as an outsider.’* P22, Male, Tujia Ethnic Group

Some of the participants reported that they often questioned themselves about why they cannot exist as both an ethnic migrant and a resident of the city. Household registration played an important role in bicultural identity of the migrants. The fact that they do not possess a local household registration in the city and considering the high housing prices most of the participants might never be able to have a household registration of their destination city which exacerbated their confusion about being a part of the city. However, it did not prevent them from having some degree of belongingness to the city.

‘*I have lived and worked in this city for a few years now. I sometimes feel like I belong here because I have lived here more than I lived in my own home since past few years. But then I am reminded that I cannot afford to buy a house here and might never be able to change my hukou (household registration) from my hometown to here. So, the reality is that I am an ethnic person from far away village but maybe I could also be a part of this city albeit not for a lifetime*.’ P26, Male, Zangzu Ethnic Group

The excerpt articulates institutionally bounded belonging. The participant’s felt attachment (‘I sometimes feel like I belong here because I have lived here more than I lived in my own home’) collides with a structural ceiling (‘I cannot afford to buy a house here and might never be able to change my hukou’), producing the carefully qualified conclusion ‘maybe I could also be a part of this city albeit not for a lifetime’. The qualifier ‘albeit not for a lifetime’ compresses the central finding of this theme: bicultural belonging develops experientially but is rationed institutionally. Identity negotiation here does not fail psychologically; it is truncated by the registration system, which converts emergent belonging into a temporary license rather than a settled status.

Bicultural identity was one of the few positive themes supporting social integration of the ethnic migrant youths which emerged from the data of the present study. Although ethnic migrant youths faced a number of challenges and negative experiences in destination cities, they developed some degree of sense of belonging over time as they adapted.

The data show that social integration is not an immediate outcome of migration but a gradual process that takes time. Participants who had lived in the city for shorter periods (3–4 years) reported stronger feelings of alienation and more frequent experiences of discrimination. Those with longer residence (5–7 years) showed greater bicultural identity integration and comfort in urban environments. For instance, P24, who had lived in the city for four years expressed growing comfort: *I have started feeling comfortable being here. I think I can definitely choose to live and work here for a few more years.* This shows that while ethnic identity retention remains strong across all timeframes, the comfort and integration in the urban environment increases with time.

### Gendered differences in ethnic identity and integration

The data reveal gendered differences in how ethnic identity intersected with social integration experiences. Female participants reported more emotionally intense responses to host-community treatment. The female participants from all three ethnic groups mentioned feeling that their ethnic identity was objectified.

*When I moved to the city the only job I got was to dress in ethnic attire and jewelry and greet customers. The women from my ethnic group wear elaborate silver headpieces and jewelry. Customers would touch me without asking. They were probably admiring me but I felt like I became a showpiece or an exhibit.* P29, Female, Tujia Ethnic Group

For the women from ethnic groups their ethnic identity often became a commodified and objectified element of urban labor market. Women also reported that their prospects of employment depended on their look and appearance which meant women experience their identity as hyper-visible and externally defined. In such a context, proximity to members of their own ethnicity provided support.

*I feel kind of freedom when I am with my villagers. It is probably the freedom of not being constantly judged*. P5, Female, Miao ethnic group

Some female participants described uncomfortable encounters in public spaces that were based on their appearance.

*It feels somehow hurtful when I sit in the bus or metro but the person next to me will move to another seat.* P22, Female, Zangzu ethnic group

Male participants, however, emphasized self-categorization, confidence, employment navigation and bicultural belonging without the same emotional intensity. Ethnic identity for men operate through network-based solidarity such as labor recruitment through co-ethnic ties, shared housing and informal economic circuits. Their identity is less publicly performed but still structurally consequential. This suggests that ethnic identity functions differently for female and male migrant youths in their social integration process.

## Discussion

The findings reveal a consistent pattern: ethnic self-identity functions as a two-sided mechanism in which protective and boundary functions are connected rather than opposed. Participants across all three ethnic groups described ethnic identity as simultaneously a psychological buffer against discrimination and a social boundary that complicates host-community integration. Upon their entry into the host communities of urban areas, migrants actively renegotiate to build their social identities of which ethnic self-identity remains an integral part. Participants interpreted the outcomes of their identity negotiation as shaped by two interacting influences which are attributes of ethnic migrant youth themselves and reaction of the host community towards the migrants [35,36]. The factors which exerts strong influence on self-identity of ethnic migrant youths are language, religious beliefs (especially for participants from Xizang), ethnic practices, and human and social capital (Verkuyten & Martinovic [37]. It is important to note that accounts of host community attitudes reflect participant’s subjective perception rather than objective measurements, as this study did not include host-community interviews. Participants consistently reported that they perceived urban host communities as responding to them in ways that affected their identity and integration. They described experiences of general attitudes, official migrant related policies, and limited access to public services as factors that shaped their identity negotiation process.

The five related factors identified in the results can be understood through theories outlined earlier. Ecological systems theory [20] shows ethnic self-categorization and language proficiency shape daily interactions at micro level, while perceived discrimination reflects broader influences such as labour-market structures and Hukou policy. Our findings extend ecological systems theory by showing that ethnic identity operates as a mediating mechanism between structural constraints and individual outcomes, a role not previously theorized in the migrant integration literature. In our participant’s accounts, identity negotiation was not merely an individual psychological process but an active interpretation of structural condition. Ethnic identity in migrant integration research should be conceptualized not only as an individual-level variable but as a mediating process that translates structural constraints into subjective experiences, suggesting that ecological analyses of migration must incorporate interpretative identity work as a meso-level mechanism.

Social identity theory [23] explains why participants maintain strong ethnic boundaries: ethnic self-categorization gives them positive identity and buffers against negative evaluations from host communities. Our findings challenge the unidimensional view of ethnic identity as either barrier or resource by demonstrating that the same identity processes simultaneously serve protective and boundary functions. Participants did not choose between ethnic belonging and host community integration, rather, the very processes that sustained their dignity in the face of discrimination also marked social distance from host communities. The protective-boundary paradox suggest that social identity theory’s in-group/out-group framework must account for contexts where positive identity and social distance are co-produced rather than traded off, particularly in structurally segregated migration setting.

We used Berry’s acculturation typology as an analytical tool to understand patterns in the data, not as a fixed classification system [24]. The dominant pattern among participants corresponded to segregation, strong ethnic identity retention with limited host-community contact. However, this pattern was not uniform. Several participants expressed forms of bicultural belonging or partial attachment to host communities indicating that acculturation positions are fluid and situationally contingent rather than fixed. According to Allport’s [38] conditions and Pettigrew and Tropp’s [39] meta-analysis, contact reduces prejudice only when equal status, common goals, inter-group cooperation, and authority endorsement are present. In our participants’ accounts, barely any of these conditions were present: there was a large status distance between locals and migrants, most participants worked in low-skill ethnic-niche jobs through co-ethnic referrals, super-ordinate goals were absent due to the ‘earn and return’ orientation, and Hukou restrictions signaled institutional indifference toward integration. Acculturation outcomes are not merely categorical positions but actively constructed through identity work that responds to perceived structural opportunities and constraints. Future acculturation research should incorporate process-oriented measures of identity negotiation alongside categorical typologies. We identify a reverse causal pathway from anticipated discrimination to identity-based social withdrawal that precedes contact reduction. This suggests that structural contact interventions alone cannot break the segregation cycle without simultaneously addressing institutional barriers that produce anticipatory mistrust.

The exposure to mainstream culture in urban areas do not erase ethnic identity but sets the ground for renegotiation of the ethnic migrant’s identity. The results show that migrants have retained a high level of ethnic identity which reflects Shao and Zhang’s study on Zangzu migrants in Chengdu and Beijing [18]. The ethnic migrants affirm their ethnic identity not only to address the challenges they face in host communities but also to maintain a sense of belonging to a community where they find themselves represented [40]. The participants of all three ethnic groups strongly retained their ethnic identity, had noticeably minimal contact with members of host communities and did not have much role in the local community activities. However, on a positive side the ethnic migrants did acknowledge that given relatively secluded lifestyle they had among the homogenous communities of their hometown, exposure to mainstream host communities of urban areas gave them a better and more critical perspective about their ethnic identity. Many participants expressed that migrating to urban areas have broadened the horizon of their insights on how they are perceived as an ethnic migrant in the larger society. Consequently, ethnic self-identity operates simultaneously as a psychological buffer against discrimination [41] and as a symbolic boundary that rationalizes low investment in long-term urban membership.

The findings must be understood within the specific structural context of Chinese rural-to-urban migration. The Hukou system creates a legally divided urban population in which ethnic migrants lack permanent residency rights, restricting access to public services, affordable housing, and quality education for children. Labor-market segmentation channels ethnic minority migrants into low-skill, precarious, ethnic-niche occupations that limit both economic advancement and workplace contact with host communities. Spatial segregation in rental housing and migrant areas physically separates ethnic migrants from host communities, limiting informal inter-ethnic interaction. These structural conditions are not secondary background factors but primary determinants of the field within which ethnic identity is negotiated. Any interpretation of ethnic identity’s role in integration that does not center these structural constraints risks giving too much weight to psychological processes while understating the institutionalized exclusion that shapes migrants’ life chances. Policy must therefore address structural barriers first; identity-based interventions alone cannot achieve integration without Hukou reform, anti-discrimination enforcement, and housing desegregation.

These findings further connect with international research on migration and urban place-identity. Studies on migration and city image [42] show that migrants’ urban integration is shaped not only by structural opportunities but by how they perceive and emotionally relate to the urban environment. Similarly, research on home in multi-ethnic settings [43] shows that migrants often build mixed senses of belonging that connect them to both home communities and destination cities. The contrast between rural settlements and urban development areas [44] further shows why ethnic identity maintenance in cities is not merely nostalgic but an active negotiation between lost place attachments and new urban identifications.

### Limitations

This study has several limitations. Because this study is qualitative, the generalizability of its results may be limited; nevertheless, it offers valuable insights into how components of ethnic self-identity shape social integration in destination cities. Although the researcher tried to be as inclusive as possible regarding participation of various ethnic groups in this study but owing to various limitations, it was only possible to contact participants from Miao, Tujia and Zangzu ethnic groups. The sample was limited to three ethnic groups in one city (Wuhan), which limits generalizability to other ethnic groups and cities. Different ethnic groups might have different backgrounds and experience of belonging to particular ethnic group but when they migrate to a particular city, they are consolidated in the same bracket of being ethnic migrants or *waidi ren* (outsiders). Furthermore, the study focused on social integration of ethnic migrant youths. In line with the demographic conventions, the term ‘youth migrant’ in this study refers to individuals aged 15 to 29 years. All participants who were interviewed were 18 years of age or older (range 18-26 years; See Table 3). The experience of ethnic migrants who belong to a different or more advanced age group might not be represented by this study. Moreover, the participants in this study had all migrated in past 5 years. The results of this study might not reflect the realities of the ethnic migrants who migrated to urban areas for a longer period. All participants had no more than high school education, which shaped their employment trajectories and housing conditions. This educational profile reflects structural educational differences in ethnic minority regions but also means findings may not apply to ethnic migrant youths with higher education. The study did not include host-community perspectives, so claims about host-community attitudes reflect participants’ subjective perceptions rather than objective measurement. The cross-ethnic comparison required a larger sample than typical IPA studies, which trades some case depth for breadth. Future research should include more ethnic groups, different cities, host-community interviews, and participants with varied educational backgrounds. However, this study provides a valuable perspective on how ethnic identity impacts or drives social integration for ethnic migrant youths. These limitations do not invalidate the insights gained from the study but delineate the boundaries within which the findings should be read and highlight the importance of mixed-methods and longitudinal designs as necessary steps in future research.

### Implications

It is unlikely that the migration trend of ethnic youth would see any decline in foreseeable future as they venture out of their ethnic enclaves in search of better economic opportunities seeking to uplift their lifestyle. Hence, it is imperative for cities and urban areas to accommodate the needs of ethnic migrant youths which would help them make an easier transition and subsequently integration.

Implications for policy and local government: The findings generate four policy implications, each grounded in participants’ accounts. (1) *Bilingual capacity, not Mandarin assimilation:* Because participants experienced ethnic language as both a necessary tool for integration (Mandarin for employment) and a protective resource (ethnic language for group solidarity), language policy should support bilingual capacity rather than prioritizing Mandarin assimilation. (2) *Structural discrimination intervention:* Because discrimination was experienced in everyday practices (public transport seating, workplace stereotypes), awareness campaigns alone are insufficient, structural interventions addressing spatial segregation and workplace discrimination are needed. (3) *Temporary migration realities:* Because participants planned to return to hometowns, integration policy should address temporary and circular migration rather than assuming permanent settlement. (4) *Cultural practice continuity:* Because ethnic cultural practices were disrupted by urban context absence, policy should support culturally specific spaces and events that enable genuine practice continuity, not merely symbolic celebration.

Municipal councils and local government should create institutional conditions that facilitate integration. This includes accelerating Hukou reform to reduce registration-based barriers that restrict ethnic migrant’s access to public services, healthcare, education for children, and housing subsidies [5]. Local governments should implement anti-discrimination policies in employment and housing. They could also establish migrant resource centers that provide legal aid, employment consultation and language classes where required.

Implication for host communities: Host communities benefit from understanding ethnic migrant’s experiences and reducing prejudice. Community awareness programs and intercultural exchange events can foster mutual understanding and reduce discrimination. Food festivals, cultural exhibitions and community cultural performance programs can be some practical ways of addressing the gap between members of ethnic migrant and host communities.

Implications for social workers and minority advocates: Social workers and minority group advocates can use these findings to design support programs that address ethnic migrant’s specific needs. This includes mental health support for those affected by discrimination and cultural preservation programs. The social workers could refer to Chinese Government’s Healthy China 2030 strategy and the National Social Psychological Service System Construction Policy which extends psychological support provisions to migrants in urban areas [45].

Implications for academia: Future research should examine host-community perspectives, include more ethnic groups, and explore the long-term integration outcomes of ethnic migrant youths in different urban contexts.

## Conclusion

This study examined how ethnic self-identity shape the social integration process of ethnic migrant youths in destination cities in China. Through Interpretative Phenomenological Analysis of Miao, Tujia and Zangzu migrant in Wuhan, the study found that ethnic self-identity functions as a two-sided mechanism which simultaneously provides psychological protection against discrimination and sustains ethnic group cohesion, while also marking social boundaries that complicate host-community integration. The dominant pattern among participants showed strong ethnic identity retention with limited host-community contact,a position consistent with Berry’s segregation quadrant, though a minority demonstrated bicultural integration. These findings are specific to the structural context of Wuhan and the three ethnic groups studied, and caution is warranted against generalizing to other destination cities and ethnic populations.

This study offers three theoretical contributions. First, we extend Berry’s acculturation framework by showing that segregation is not merely a category but an outcome that migrants actively construct through identity processes. Participants described responding to limited opportunities by strengthening their ethnic identity, which they experienced as contributing to segregation. This suggests acculturation research should incorporate process-oriented, experiential accounts alongside categorical measurement. Second, we extend contact theory by identifying a reverse pathway: anticipated discrimination motivates identity-based social withdrawal, which reduces contact opportunities and reinforces prejudice, a cycle that structural contact interventions alone cannot break without addressing institutional barriers. Third, we propose that ethnic identity functions as a protective-boundary mechanism that combines psychological and sociological dimensions of migrant integration, showing how the same identity processes simultaneously buffer against discrimination and mark social distance from host communities. For rural-to-urban ethnic minority migrants in China, this mechanism is shaped by the Hukou system and ethnic-niche labour markets, a structural dynamic not well explained in Western acculturation models. Moreover, the ‘earn and return’ orientation among participants shows that ethnic identity functions as a resource for maintaining home connections rather than a static acculturation outcome. This challenges the assumption that migrants always seek permanent settlement.

These findings carry implications for policy and practice. Hukou reform remains essential, as registration-based barriers restrict ethnic migrants’ access to public services, affordable housing, and quality education. Anti-discrimination enforcement in employment and housing should accompany awareness campaigns, because participants experienced discrimination in everyday practices such as public transport seating and workplace stereotyping. Language policy should support bilingual capacity rather than Mandarin assimilation alone, because participants experienced ethnic language as both an employment tool and a protective group resource. Migrant resource centres providing legal aid, employment consultation, and culturally specific programming could address the absence of ethnic community institutions that participants identified as a barrier to practice continuity. Finally, integration policy should account for temporary and circular migration realities rather than assuming permanent settlement intent.

The study opens several avenues for future research. First, future researchers could adopt multi-perspective designs that include hos-community voices alongside migrant’s accounts which would help validate the subjective perceptions reported here. Second, the longitudinal approach would be valuable for tracking how migrants’ acculturation positions change over time, especially as structural conditions such as Hukou policy continue to evolve. Third, comparative studies across different Chinese cities and with other ethnic minority groups would clarify whether the protective- boundary mechanisms identified in this study appears in other settings as well.
